# Correlations between Circulating and Tumor-Infiltrating CD4^+^ T Cell Subsets with Immune Checkpoints in Colorectal Cancer

**DOI:** 10.3390/vaccines10040538

**Published:** 2022-03-30

**Authors:** Mohammad A. Al-Mterin, Khaled Murshed, Eyad Elkord

**Affiliations:** 1 Natural and Medical Sciences Research Center, University of Nizwa, Nizwa 616, Oman; mohammed.mterin@unizwa.edu.om; 2 Department of Pathology, Hamad Medical Corporation, Doha 5207, Qatar; kmurshed@hamad.qa; 3 Biomedical Research Center, School of Science, Engineering and Environment, University of Salford, Manchester M5 4WT, UK

**Keywords:** correlation, FoxP3, Helios, Tregs, immune checkpoints, CRC

## Abstract

T regulatory cells (Tregs) play different roles in the regulation of anti-tumor immunity in colorectal cancer (CRC), depending on the presence of different Treg subsets. We investigated correlations between different CD4^+^ Treg/T cell subsets in CRC patients with immune checkpoint-expressing CD4^+^ T cells. Positive correlations were observed between levels of different immune checkpoint-expressing CD4^+^ T cells, including PD-1, TIM-3, LAG-3, and CTLA-4 with FoxP3^+^ Tregs, Helios^+^ T cells, FoxP3^+^Helios^+^ Tregs, and FoxP3^+^Helios^−^ Tregs in the tumor microenvironment (TME). However, negative correlations were observed between levels of these immune checkpoint-expressing CD4^+^ T with FoxP3^−^Helios^−^ T cells in the TME. These correlations in the TME highlight the role of cancer cells in the upregulation of IC-expressing Tregs. Additionally, positive correlations were observed between levels of FoxP3^+^ Tregs, Helios^+^ T cells, FoxP3^+^Helios^+^ Tregs, and FoxP3^+^Helios^−^ Tregs and levels of CD4^+^CTLA-4^+^ T cells and CD4^+^PD-1^+^ T cells in peripheral blood mononuclear cells (PBMCs) and normal tissue-infiltrating lymphocytes (NILs). These observations suggest that CTLA-4 and PD-1 expressions on CD4^+^ T cell subsets are not induced only by the TME. This is the first study to investigate the correlations of different FoxP3^+/−^Helios^+/−^ T cell subsets with immune checkpoint-expressing CD4^+^ T cells in CRC patients. Our data demonstrated strong correlations between FoxP3^+/^Helios^+/−^ Tregs but not FoxP3^−^Helios^+/−^ non-Tregs and multiple immune checkpoints, especially in the TME, providing a rationale for targeting these cells with highly immunosuppressive characteristics. Understanding the correlations between different immune checkpoints and Treg/T cell subsets in cancer patients could improve our knowledge of the underlying mechanisms of Treg-mediated immunosuppression in cancer.

## 1. Introduction

Colorectal cancer (CRC) is the third most common cancer and the second most fatal malignancy in the world [1]. The gastrointestinal tract is susceptible to persistent immune responses, leading to chronic intestinal inflammation, which has a role in the development of cancer, particularly via the secretion of inflammatory cytokines [2]. Tumor cells have a close interaction with stromal cells in the tumor microenvironment (TME) [3]. The composition of the TME during CRC tumorigenesis is found to be different from normal intestinal stroma [4]. It is now clear that the TME has an essential role in tumor progression, and it offers an ideal environment for the discovery of therapeutic targets [4]. Immune checkpoints (ICs) and their ligands are commonly upregulated in the TME of several cancers, leading to the suppression of anti-tumor immunity [5]. Tumors could modulate the expression of certain ICs as a substantial mechanism of immunological resistance against tumor cells [6,7]. T regulatory cells (Tregs) are immunosuppressive cells that are present in different subsets and serve key functions in the maintenance of immunological homeostasis, self-tolerance, and regulation of cancer immunity [8]. In different types of cancer, high levels of Treg infiltration into tumors are usually correlated with poor clinical outcomes [9]. However, the role of Tregs is controversial in CRC. Certain studies have shown that tumor-infiltrating FoxP3^+^ Tregs are associated with a better prognosis in CRC patients [7,10,11,12]. Conversely, Betts et al., found that high levels of Tregs contribute to disease progression in CRC patients [13]. Helios serves as a marker for T cell activation and proliferation and its expression is required for the maintenance of Treg inhibitory function [14]. In CRC, Helios mRNA levels were shown to be higher in tumor tissue in advanced stages, suggesting their potential effects in CRC progression [15]. We have recently reported that some inhibitory ICs, including programmed cell death-1 (PD-1), cytotoxic T lymphocyte-associated antigen (CTLA-4), T cell immunoglobulin and mucin domain-containing protein-3 (TIM-3), and lymphocyte-activation gene-3 (LAG-3) are upregulated on CD4^+^ T cells and play roles in CRC progression [7]. In this study, we investigated potential correlations between levels of different Treg subsets and CD4^+^ T cells expressing different immune checkpoints.

## 2. Materials and Methods

### 2.1. Patients and Samples

The clinical and pathological characteristics of patients involved in this study are as described in Table 1. Thirty-two patients were eligible and included in the correlation analyses reported in this study. Normal colon tissues and tumor tissues were obtained from 22 of these 32 patients. The study was carried out with ethical approval from the Hamad Medical Corporation, Doha, Qatar (protocol no. MRC-02-18-012). All patients were treatment-naive prior to surgery, and they gave written informed consent prior to sample collection. PBMCs were isolated from fresh blood by density-gradient centrifugation, and cells were suspended in freezing media and stored in liquid nitrogen for further analyses. Cells were isolated from normal and tumor tissues by mechanical disaggregation. Tissue disaggregation was performed on a gentleMACS dissociator (Miltenyi Biotech, Bergisch Gladbach, Germany) without using enzymes. Afterwards, the single-cell suspension was washed and stained for flow cytometric analyses.

### 2.2. Multi-Parametric Flow Cytometry

No additional experiments were performed in this study, and flow cytometry data for different CD4^+^ T cell subsets and immune checkpoint expressions were collected. Immune staining and flow cytometry analyses were carried out as per our previously published article [7]. Briefly, PBMC and cells isolated from tissues were washed and re-suspended in flow cytometry staining buffer. Then, Fc receptors (FcR) were first blocked using FcR Blocker. Afterwards, cells were stained with specific cell surface antibodies. All data were obtained using BD FACSDiva software (BD Biosciences) and analyzed with FlowJo V10 software (FlowJo, Ashland, Wilmington, NC, USA).

### 2.3. Statistical Analyses

Correlation analyses were performed using GraphPad Prism 9 software (GraphPad Software, San Diego, CA, USA. The Shapiro-Wilk test was used to analyze the normality of datasets. The Pearson correlation test was utilized for normally distributed data, and Spearman’s rank correlation test was used for non-normally distributed data. A *p*-value of ≤0.05 was considered to be statistically significant.

## 3. Results

### 3.1. Correlations between Levels of FoxP3^+^ Tregs and Helios^+^ T cells with Levels of IC-Expressing CD4^+^ T Cells

**Figure 1:**
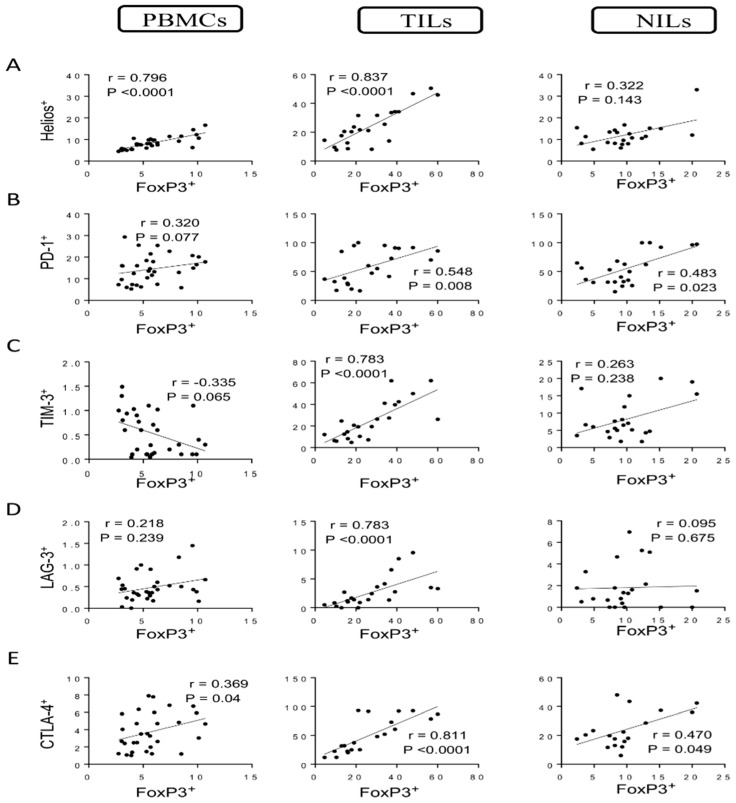
Correlations between levels of FoxP3 and immune checkpoints in CD4^+^ T cells in CRC patients. Correlations between levels of CD4^+^FoxP3^+^ Tregs with Helios^+^ (**A**), PD-1^+^ (**B**), TIM-3^+^ (**C**), LAG-3^+^ (**D**), and CTLA-4^+^ (**E**) in PBMCs, TILs, and NILs.

Tregs are a significant subgroup of CD4^+^ T cells that are characterized by high levels of the interleukin-2 receptor alpha chain (CD25) and the transcription factor FoxP3 [16]. Helios is a transcription factor that modulates FoxP3^+^ Treg functional stability and is needed for the inhibitory action of these cells [14,17,18,19]. We have previously generated a flow cytometric analysis with representative plots and determined levels of different CD4^+^ T cell subsets in PBMCs, NILs, and tumor-infiltrating lymphocytes (TILs) of CRC patients [7]. In this study, we further identified the correlation between levels of FoxP3^+^ Tregs with Helios^+^ T cells in circulation, normal tissues, and TME in CRC patients. Strong correlations were found between levels of FoxP3^+^ Tregs and Helios^+^ T cells in circulation and tumor tissues (correlation coefficient *r =* 0. 0.796, *p* < 0.0001; *r* = 0.837, *p* < 0.0001, respectively) but not in normal tissues (Figure 1A). We investigated, for the first time, the correlations between CD4^+^ Treg subsets with certain IC-expressing CD4^+^ T cells in CRC patients. We found a moderate correlation between levels of FoxP3^+^ Tregs and CD4^+^PD-1^+^ T cells in PBMCs and normal tissues (*r* = 0.370, *p* = 0.037; *r* = 0.483, *p* = 0.023, respectively). Interestingly, a significant correlation was observed in tumor tissues between levels of FoxP3^+^ Tregs and CD4^+^PD-1^+^ T cells (*r* = 0.548, *p* = 0.008) (Figure 1B). Moreover, a moderate negative correlation was found between levels of FoxP3^+^ Tregs and CD4^+^TIM-3^+^ T cells in circulation (*r* = −0.335, *p* = 0.065) (Figure 1C). Conversely, a strong positive correlation (*r* = 0.783, *p* < 0.0001) was observed between levels of FoxP3^+^ Tregs and CD4^+^TIM-3^+^ T cells in tumor tissues but not in normal tissues (Figure 1C). Furthermore, a strong positive correlation (*r =* 0.783, *p* < 0.0001) was observed between levels of FoxP3^+^ Tregs and CD4^+^LAG-3^+^ T cells in tumor tissues but not in PBMCs and NILs (Figure 1D). Moderate correlations were observed between levels of FoxP3^+^ Tregs and CD4^+^CTLA-4^+^ T cells in PBMCs and NILs (*r* = 0.469, *p* = 0.04; *r* = 0.470, *p* = 0.049, respectively) (Figure 1E), while a strong positive correlation was found between levels of FoxP3^+^ Tregs and CD4^+^CTLA-4^+^ T cells (*r* = 0.811, *p* < 0.0001) in tumor tissues (Figure 1E).

**Figure 2:**
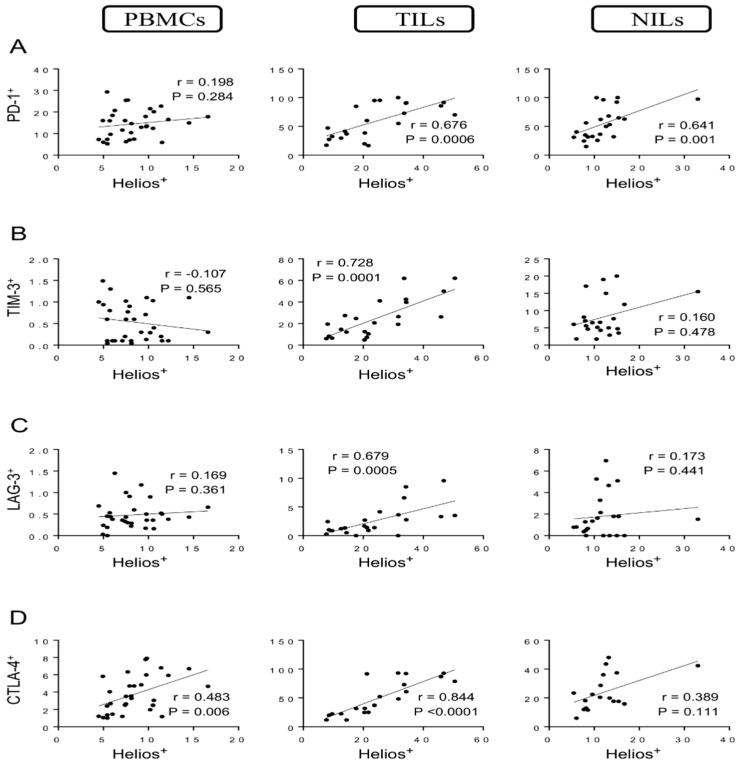
Correlations between levels of Helios and immune checkpoints in CD4^+^ T cells in CRC patients. Correlations between levels of CD4^+^Helios^+^ T cells with PD-1^+^ (**A**), TIM-3^+^ (**B**), LAG-3+ (**C**), and CTLA-4^+^ (**D**) in PBMCs, TILs, and NILs.

Strong correlations were observed between levels of Helios^+^ T cells and CD4^+^PD-1^+^ T cells in TILs and NILs (*r* = 0.676, *p* = 0.0006; *r* = 0.641, *p* = 0.001, respectively) but not in PBMCs (Figure 2A). Indeed, a strong positive correlation was found between levels of Helios^+^ T cells and CD4^+^TIM-3^+^ T cells in TILs (*r* = 0.728, *p* = 0.0001) but not in PBMCs and NILs (Figure 2B). Similarly, a strong positive correlation was found between levels of Helios^+^ T cells and CD4^+^LAG-3^+^ T cells in TME (*r* = 0.679, *p* = 0.0005) but not in PBMCs and NILs (Figure 2C). Furthermore, the following correlations between levels of Helios^+^ T cells and CD4^+^CTLA-4^+^ T cells were observed: a strong positive correlation in tumor tissues (*r* = 0.844, *p* < 0.0001), a moderate correlation in circulation (*r* = 0. 483, *p* = 0.006), and a non-significant moderate correlation in normal tissues (*r* = 0.389, *p* = 0.111) (Figure 2D).

### 3.2. Correlations between Levels of Different FoxP3^+/−^Helios^+/−^ Cell Subsets and IC-Expressing CD4^+^ T Cells

**Figure 3:**
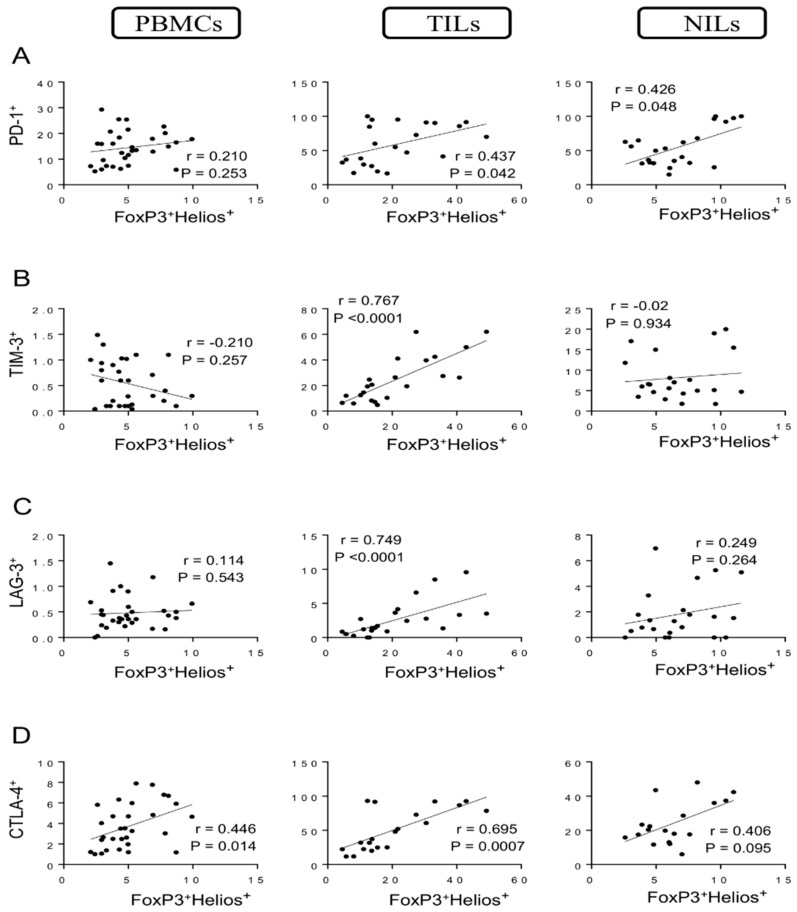
Correlations between levels of FoxP3^+^Helios^+^ and immune checkpoints in CD4^+^ T cells in CRC patients. Correlations between levels of CD4^+^FoxP3^+^Helios^+^ Tregs with PD-1^+^ (**A**), TIM-3^+^ (**B**), LAG-3^+^ (**C**), and CTLA-4^+^ (**D**) in PBMCs, TILs, and NILs.

We went further and identified the correlations between different FoxP3^+/−^Helios^+/−^ T cell subsets with specific IC-expressing CD4^+^ T cells. We found a moderate correlation between levels of FoxP3^+^Helios^+^ Tregs and CD4^+^PD-1^+^ T cells in TILs and normal tissues (*r* = 0.437, *p* = 0.042; *r* = 0.426, *p* = 0.048, respectively) but not in PBMCs (Figure 3A). Moreover, strong positive correlations were observed between levels of FoxP3^+^Helios^+^ Tregs with CD4^+^TIM-3^+^ T cells and CD4^+^LAG-3^+^ T cells (*r =* 0.767, *p* < 0.0001; *r* = 0.749, *p* < 0.0001, respectively) in tumor tissues but not in circulation or normal tissues (Figure 3B,C). Furthermore, a strong positive correlation was found between levels of FoxP3^+^Helios^+^ Tregs and CD4^+^CTLA-4^+^ T cells (*r* = 0.695, *p* = 0.0007) in tumor tissues (Figure 3D). Indeed, moderate correlations were observed between levels of FoxP3^+^Helios^+^ Tregs and CD4^+^CTLA-4^+^ T cells in PBMCs and NILs (*r* = 0.446, *p* = 0.014; *r* = 0.406, *p* = 0.095, respectively) (Figure 3D).

**Figure 4:**
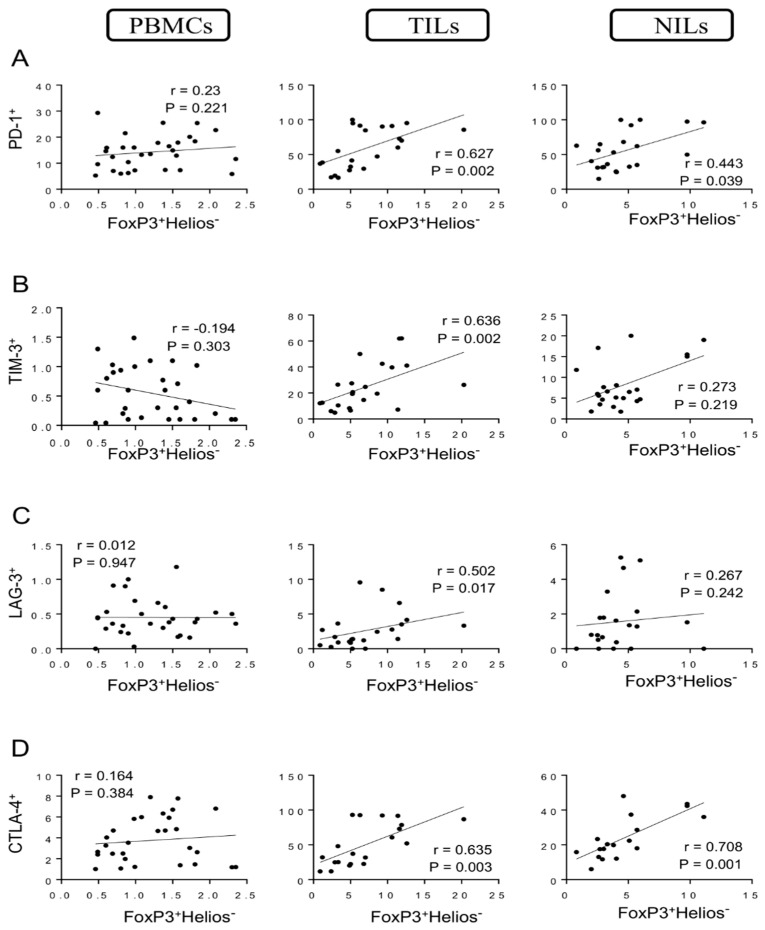
Correlations between levels of FoxP3^+^Helios^−^ and immune checkpoints in CD4^+^ T cells in CRC patients. Correlations between levels of CD4^+^FoxP3^+^Helios^−^ Tregs with PD-1^+^ (**A**), TIM-3^+^ (**B**), LAG-3^+^ (**C**), and CTLA-4^+^ (**D**) in PBMCs, TILs, and NILs.

When we investigated Tregs lacking Helios expression (FoxP3^+^Helios^−^), we found a strong positive correlation between levels of these Tregs and CD4^+^PD-1^+^ T cells (*r* = 0.627, *p* = 0.002) in tumor tissues, while moderate correlations were found between levels of these cell subsets in normal tissues (*r* = 0.443, *p* = 0.039) but not in PBMCs (Figure 4A). Furthermore, strong positive correlations were found between levels of FoxP3^+^Helios^−^ Tregs with CD4^+^TIM-3^+^ T cells and CD4^+^LAG-3^+^ T cells (*r* = 0.636, *p* = 0.002; *r* = 0.502, *p* = 0.017, respectively) in tumor tissues but not in PBMCs and NILs (Figure 4B,C). Moreover, strong positive correlations were found between levels of FoxP3^+^Helios^−^ Tregs and CD4^+^CTLA-4^+^ T cells in TILs and NILs (*r* = 0.635, *p* = 0.003; *r* = 0.708, *p* = 0.001, respectively) but not in PBMCs (Figure 4D).

**Figure 5:**
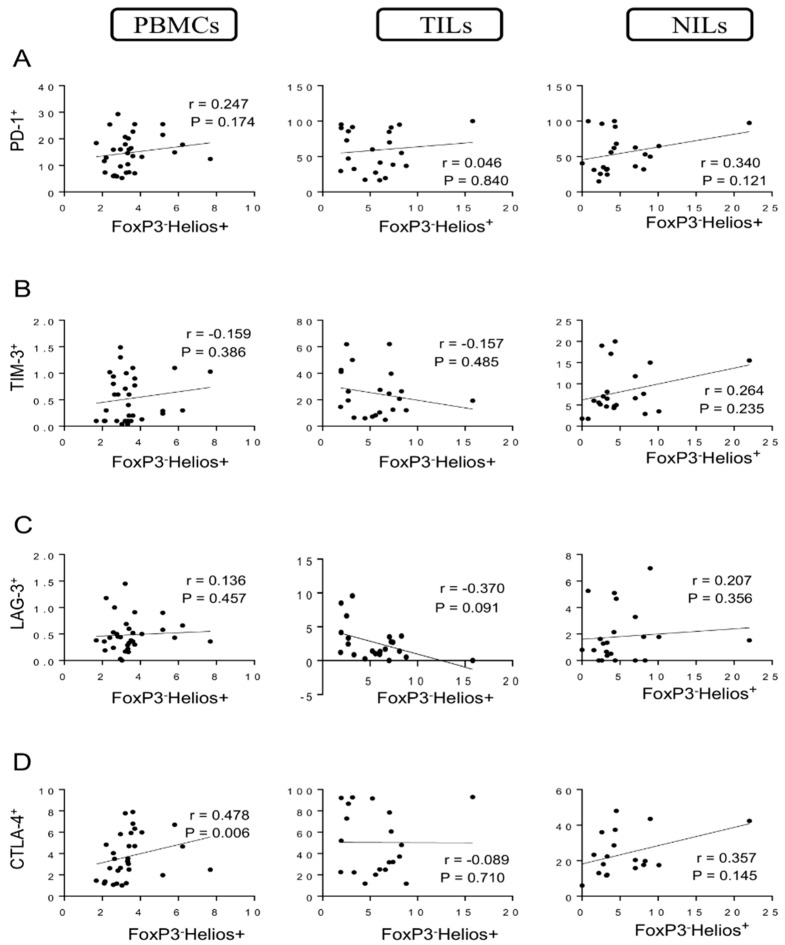
Correlations between levels of FoxP3^−^Helios^+^ and immune checkpoints in CD4^+^ T cells in CRC patients. Correlations between levels of CD4^+^FoxP3^−^Helios^+^ T cells with PD-1^+^ (**A**), TIM-3^+^ (**B**), LAG-3^+^ (**C**), and CTLA-4^+^ (**D**) in PBMCs, TILs, and NILs.

**Figure 6:**
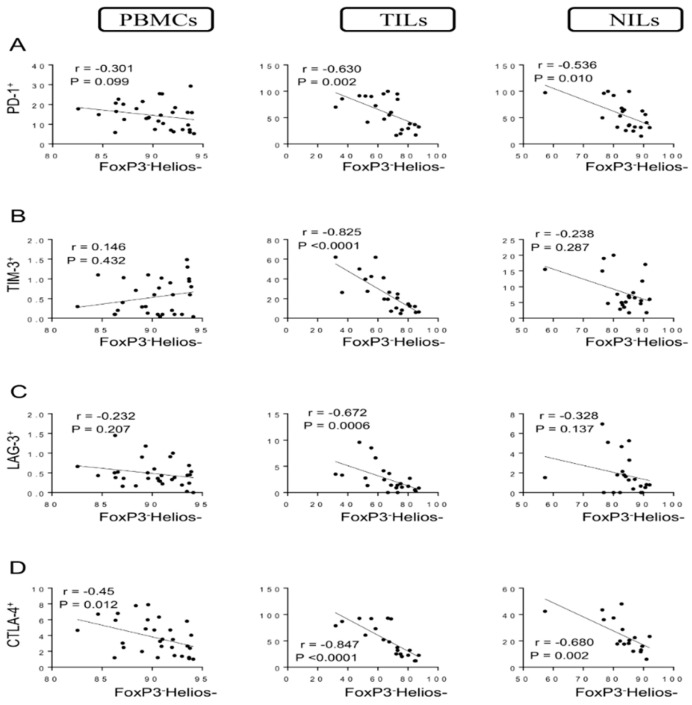
Correlations between levels of FoxP3^−^Helios^−^ and immune checkpoints in CD4^+^ T cells in CRC patients. Correlation between levels of CD4^+^FoxP3^−^Helios^−^ T cells with PD-1^+^ (**A**), TIM-3^+^ (**B**), LAG-3^+^ (**C**), and CTLA-4^+^ (**D**) in PBMCs, TILs, and NILs.

We then investigated these correlations in FoxP3^−^ non-Tregs. Interestingly, there were no correlations between levels of FoxP3^−^Helios^+^ T cells and CD4^+^ T cells expressing PD-1, TIM-3, and LAG-3 in PBMCs, TILs, and NILs (Figure 5A–C). However, a significant moderate correlation was observed between levels of FoxP3^−^Helios^+^ T cells and CD4^+^CTLA-4^+^ T cells in circulation (*r* = 0.478, *p* = 0.006) but not in TILs and NILs (Figure 5D). Additionally, negative correlations were observed between levels of FoxP3^−^Helios^−^ T cells and CD4^+^PD-1^+^ T cells in PBMCs, TILs, and NILs (*r* = −0.301, *p* = 0.099; *r* = −0.630, *p* = 0.002; *r* = −0.536, *p* = 0.010, respectively) (Figure 6A). Moreover, strong negative correlations were observed between levels of FoxP3^−^Helios^−^ T cells with CD4^+^TIM-3^+^ T cells and CD4^+^LAG-3^+^ T cells in tumor tissues (*r* = −0.825, *p* < 0.0001; *r* = −0.672, *p* = 0.0006, respectively) but not in PBMCs and NILs (Figure 6B,C). Finally, strong negative correlations were observed between levels of FoxP3^−^Helios^−^ T cells and CD4^+^CTLA-4^+^ T cells in PBMCs, TILs, and NILs (*r* = −0.45, *p* = 0.012; *r* = −0.847, *p* < 0.0001; *r* = −0.680, *p* = 0.002, respectively) (Figure 6D).

## 4. Discussion

CD4^+^ T cells may target cancer cells by modulating the tumor microenvironment [20,21]. However, the majority of CD4^+^ T cells in different tumors, including CRC, are not effector cells but predominantly T regulatory cells, expressing several IC molecules, such as PD-1, CTLA-4, TIM-3, and others [7]. High levels of Treg-related markers were observed in the TME in CRC patients, suggesting their potential effects on carcinogenesis [7,22,23]. In contrast to other solid tumors, high levels of tumor-infiltrating FoxP3^+^ Tregs were related to increased survival in CRC patients [12,24]. Moreover, a high frequency of FoxP3^+^ Tregs within the tumor leads to a promising outcome in CRC, suggesting that FoxP3^+^ Tregs are one of the most useful predictive indicators of disease prognosis in CRC patients [11]. However, it has been demonstrated that circulating Tregs are effective in suppressing antitumor immunity, leading to an adverse outcome in CRC patients [13,25]. The tumor tissues in CRC were characterized by high levels of Helios^+^ Tregs compared to PBMCs and normal colon tissues [24,26], suggesting a potential role for Helios in CRC progression [15]. Moreover, our group has recently observed a strong positive correlation between FoxP3^+^ and Helios^+^ expression in TILs and in the circulation of CRC patients [19]. Consistent with these data, we observed a strong positive correlation between levels of FoxP3^+^ Tregs and Helios^+^ CD4^+^ T cells in TILs and PBMCs, suggesting that circulating and intratumoral FoxP3^+^Tregs have higher expressions of Helios, confirming their highly activated status and immunosuppressive characteristics [27,28]. Moreover, we have shown that FoxP3^+^Helios^+^ Tregs have more immunosuppressive characteristics, such as secretion of IL-10, GARP/LAP expression, and absence of effector cytokine secretion (IFN-γ and IL-2) compared with FoxP3^+^Helios^−^ Tregs [29].

Expression of inhibitory IC receptors, including PD-1, TIM-3, LAG-3, and CTLA-4 on Tregs, is crucially important for their suppressive function [30,31,32,33]. Additionally, increased levels of intratumoral Tregs expressing ICs inhibit the activation and proliferation of cytotoxic CD8^+^ T cells and CD4^+^ effector T cells within the tumor [34]. Our recent work reported that key ICs, including CTLA-4, PD-1, LAG-3, and TIM-3, were highly expressed on CD4^+^ T cells in the TME of CRC patients [7]. PD-1 improves FoxP3 expression and the suppressive function of induced Tregs in tumor tissues [35]. In our study, strong correlations were observed between levels of CD4^+^PD-1^+^ T cells and FoxP3^+^ Tregs, Helios^+^ T cells, FoxP3^+^Helios^+^ Tregs, and FoxP3^+^Helios^−^ Tregs in TILs. Moreover, levels of CD4^+^PD-1^+^ T cells were correlated with levels of FoxP3^+^ Tregs, Helios^+^ T cells, FoxP3^+^Helios^+^ Tregs, FoxP3^+^Helios^−^ Tregs, and FoxP3^−^Helios^+^ T cells in normal tissues, indicating that PD-1 expression is not induced solely by tumor cells. Additionally, a negative correlation was observed between levels of CD4^+^PD-1^+^ T cells and FoxP3^−^Helios^−^ T cells, indicating that PD-1 expression correlates with CD4^+^ T cells expressing FoxP3 in circulation, TILs, and NILs. The upregulation of TIM-3 on CD4^+^ T cells may occur in the TME, and, in turn, the regulatory effects of these cells may contribute to the immunosuppressive environment in tumors [36]. Additionally, Gao et al. found that TIM-3 is highly upregulated on both CD4^+^ and CD8^+^ TILs from human lung cancer tissues but insignificantly expressed on PBMCs [33]. Moreover, LAG-3^+^ Tregs from colorectal cancer patients are highly suppressive and proliferative [32]. A recent study observed that upregulation of LAG-3 on tumor tissues was associated with a bad prognosis in patients with microsatellite instability-high colon cancer [37]. In our study, strong positive correlations were observed between levels of CD4^+^TIM^+^ T cells with FoxP3^+^ Tregs, Helios^+^ T cells, FoxP3^+^Helios^+^ Tregs, and FoxP3^+^Helios^−^ Tregs in TILs. However, a strong negative correlation was found between CD4^+^TIM^+^ T cells and FoxP3^−^Helios^−^ T cells. The same findings were seen in LAG-3. These correlations in TME highlight the role of the tumor in stimulating the upregulation of IC-expressing Tregs.

Jaberipour et al. found a significant correlation between CTLA-4 and FoxP3 expression in the PBMCs of breast cancer patients [38]. Moreover, Toor et al. observed that there was a high increase in levels of CD4^+^CTL-4^+^ T cells only in the PBMCs of CRC patients with advanced stages, suggesting that there is a relationship between increased levels of CTLA-4^+^ Tregs and CRC progression [7]. Importantly, Saleh et al. showed that mRNA levels of CTLA-4 in tumor tissues were increased in advanced stages of CRC [15]. In our study, strong positive correlations were observed between levels of CD4^+^CTLA-4^+^ T cells with FoxP3^+^ Tregs, Helios^+^ T cells, FoxP3^+^Helios^+^ Tregs, and FoxP3^+^Helios^−^ Tregs in TILs. In addition, CD4^+^CTLA-4^+^ T cells showed positive correlations with FoxP3^+^ Tregs, Helios^+^ T cells, FoxP3^+^Helios^+^ Tregs, FoxP3^+^Helios^−^ Tregs, and FoxP3^−^Helios^+^ in PBMCs and NILs. However, a negative correlation was observed between levels of CD4^+^CTLA-4^+^ T cells and FoxP3^−^Helios^−^ T cells in TILs, PBMCs, and NILs. These results suggest that CTLA-4 is not induced only by TME. Unfortunately, expression levels of these genes by qPCR cannot be determined because of the unavailability of patient samples in this study. Further investigations are needed to validate these findings in larger cohorts of patients. Moreover, additional experiments are required to clarify the role of these subsets in the functional modulation of Tregs in the tumor tissue and circulation of CRC patients. Moreover, it would be interesting to investigate the correlations between different Treg subsets and T cells co-expressing different immune checkpoints.

## 5. Conclusions

To date, this is the first study to investigate the correlations of different FoxP3^+/−^Helios^+/−^ T cell subsets with immune checkpoint-expressing CD4^+^ T cells in CRC patients. Important findings in this study showed that levels of FoxP3^+^Helios^+/−^ Treg subsets in the TME correlate strongly with levels of immune checkpoint-expressing CD4^+^ T cells in CRC patients. Given the low levels of FoxP3 and Helios expression in PBMCs, the correlations investigated in PBMCs should be interpreted carefully. Understanding the correlations between different immune checkpoints and Treg/T cell subsets in cancer patients could improve our knowledge of the underlying mechanisms of Treg-mediated immunosuppression in cancer.

## Figures and Tables

**Table 1:** Characteristic features of study populations.

	CRC Patients
Number	32 (22)
Age (Median)	61 (31–96)
Gender (Male: Female)	23:9
TNM stages	
I	5 (1)
II	9 (8)
III	15 (11)
IV	3 (2)
Tumor budding	
Low	11 (7)
Intermediate	11 (7)
High	10 (8)

## Data Availability

The data presented in this study are available on request from the corresponding author.
